# Hippocampal FGF-2 and BDNF overexpression attenuates epileptogenesis-associated neuroinflammation and reduces spontaneous recurrent seizures

**DOI:** 10.1186/1742-2094-7-81

**Published:** 2010-11-18

**Authors:** Roberta Bovolenta, Silvia Zucchini, Beatrice Paradiso, Donata Rodi, Flavia Merigo, Graciela Navarro Mora, Francesco Osculati, Elena Berto, Peggy Marconi, Andrea Marzola, Paolo F Fabene, Michele Simonato

**Affiliations:** 1 Section of Pharmacology, Department of Clinical and Experimental Medicine, and Neuroscience Center, University of Ferrara, Italy; 2 National Institute of Neuroscience, Italy; 3 Section of Anatomy, Department of Neurological, Neuropsychological, Morphological and Movement Sciences, University of Verona, Verona, Italy; 4 IRCSS "Bonino Pulejo", Messina, Italy; 5 Section of Microbiology, Department of Experimental and Diagnostic Medicine, University of Ferrara, Italy; 6 Section of Pathology, Department of Experimental and Diagnostic Medicine, University of Ferrara, Italy; 7 Instituto de Neurociencias, CSIC & Universidad Miguel Hernández, San Juan de Alicante, Spain

## Abstract

Under certain experimental conditions, neurotrophic factors may reduce epileptogenesis. We have previously reported that local, intrahippocampal supplementation of fibroblast growth factor-2 (FGF-2) and brain-derived neurotrophic factor (BDNF) increases neurogenesis, reduces neuronal loss, and reduces the occurrence of spontaneous seizures in a model of damage-associated epilepsy. Here, we asked if these possibly anti-epileptogenic effects might involve anti-inflammatory mechanisms. Thus, we used a Herpes-based vector to supplement FGF-2 and BDNF in rat hippocampus after pilocarpine-induced status epilepticus that established an epileptogenic lesion. This model causes intense neuroinflammation, especially in the phase that precedes the occurrence of spontaneous seizures. The supplementation of FGF-2 and BDNF attenuated various parameters of inflammation, including astrocytosis, microcytosis and IL-1β expression. The effect appeared to be most prominent on IL-1β, whose expression was almost completely prevented. Further studies will be needed to elucidate the molecular mechanism(s) for these effects, and for that on IL-1β in particular. Nonetheless, the concept that neurotrophic factors affect neuroinflammation in vivo may be highly relevant for the understanding of the epileptogenic process.

## Findings

Many acquired epilepsies have an identifiable cause, such as head trauma, an episode of status epilepticus (SE), stroke, or brain infection [1]. It is thought that these insults set in motion a cascade of neurobiological events that, in time, will lead to the occurrence of spontaneous seizures and to the diagnosis of epilepsy. This phenomenon is termed "epileptogenesis". Conventional "antiepileptic" agents may exert symptomatic effects on seizures but do not interfere with epileptogenic processes [2].

In principle, understanding the molecular mechanisms underlying the cellular alterations occurring during epileptogenesis (which include cell death, axonal and dendritic plasticity, neurogenesis, neuroinflammation and functional alterations in ion channel and synaptic properties) should allow development of effective agents. In this respect, neurotrophic factors (NTFs) appear to be strong candidates, because an extensive literature demonstrates their involvement in many of the cellular alterations associated with epileptogenesis [3]: not only do their trophic effects suggest involvement in cell death, neurogenesis and axonal sprouting, but they also exert functional effects at the synaptic level, with distinct modulatory actions at excitatory and inhibitory synapses [4]. In fact, we have recently reported that viral vector-mediated supplementation of two NTFs, namely fibroblast growth factor-2 (FGF-2) and brain-derived neurotrophic factor (BDNF), in a lesioned hippocampus favors "good" neurogenesis, repairs neuronal damage and may prevent epileptogenesis [5].

Recently, central nervous system (CNS) inflammation associated with blood-brain barrier (BBB) leakage has been implicated in the progression to epilepsy [6-9], and the pro-epileptogenic role of neuroinflammation has received great attention [10-13]. However, it is still unknown if NTFs may modulate these phenomena.

The aim of this study was to analyze if NTFs-induced anti-epileptogenic effects may involve anti-inflammatory mechanisms. We used a previously generated viral vector [5] to supplement FGF-2 and BDNF in the hippocampus after pilocarpine-induced SE that established an epileptogenic lesion. We decided to start investigating IL-1β expression and glial activation in controls and inoculated animals. An increase in inflammatory cytokines, IL-1β in particular, has been reported in the CNS and plasma in experimental models of seizures and in clinical cases of epilepsy [14]. Astrocytic and microglial activation elicited by pilocarpine-induced SE could underlie epileptogenic mechanisms [15,16] and BBB alterations [17]. A detailed description of the methods employed in this study is provided as Additional file 1.

In the pilocarpine model, an episode of SE produces, after a latent period of a few days, spontaneous recurrent seizures (SRSs), i.e. epilepsy. Pilocarpine (300 mg/kg i.p.) rapidly induced a robust convulsive SE (latency: 18 ± 2 min), which was interrupted after 2 h by administering the anticonvulsant diazepam (10 mg/kg i.p.). Based on behavioral observation and EEG recordings, the severity of SE in different animals was indistinguishable. This procedure caused damage in limbic and extra-limbic brain areas: in particular, hippocampal damage and was invariably remarkable [5]. SRSs began to occur 11 ± 1 days after SE.

Three days after SE, i.e. during latency, these lesioned animals were randomly assigned to 3 groups: one group was injected in one hippocampus with the vector expressing FGF-2 and BDNF (TH-FGF2/0-BDNF), another was injected with a control vector and the third was not treated at all (no difference was observed between these latter 2 groups in the parameters examined in this and in previous [5] studies and, therefore, they have been pooled together for statistical analysis). TH-FGF2/0-BDNF provides a short-term (about a week) increase in FGF-2 expression accompanied and followed by a slightly longer-lasting (at least 11 days) increase in BDNF expression, in the absence of significant toxicity [5]. As expected on the basis of the ability of HSV to enter nerve terminals and to be retrogradelly transported, reaching connected areas, transgene expression was bilateral, even if more robust at the site of injection for the first few days [5].

Animals were killed 7, 14 or 28 days after SE (i.e. 4, 11 or 25 days after vector inoculation) to examine possible effects on neuroinflammation. Continuous video-EEG monitoring for the occurrence, severity and duration of seizures was performed starting 7 days after SE. As previously reported [5], vector-injected rats exhibited significantly less spontaneous seizures/day compared to untreated or control vector-injected animals (Table 1). These seizures were also significantly less severe in vector-treated rats although, on average, they had the same duration in both groups (Table 1).

**Table 1:** EEG and behavioral analysis

	seizures/day	seizure score	seizure duration (s)
pilo - control vector	3.9 ± 0.6	2.4 ± 0.2	55 ± 15
pilo - FGF2-BDNF	1.2 ± 0.3**	1.5 ± 0.3*	63 ± 16

Average frequency, severity and duration of spontaneous seizures in the chronic period (14 -28 days after pilocarpine- induced SE, vectors injected 4 days after SE). Data are the means ± s.e.m. for 6 animals per group. **P < 0.01 vs. pilo-control vector; Student's t test for unpaired data. *P < 0.05 vs. pilo-control vector; Mann-Whitney U test.

**Figure 1:**
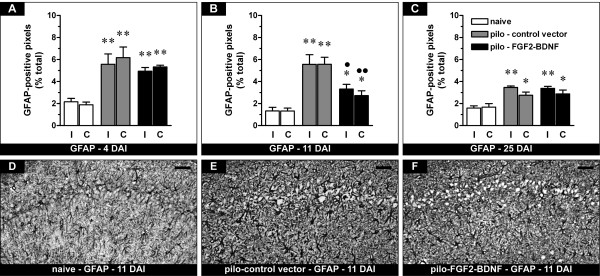
**Effects of FGF-2 and BDNF on hippocampal GFAP-positive cells (putative astrocytes)**. (A-C) Time course of alterations in GFAP expression, expressed as percent of GFAP-positive pixels in naïve rats (open bars), pilocarpine rats inoculated with the control vector (pilo - control vector, gray bars) or with the vector expressing FGF-2 and BDNF (pilo - FGF2-BDNF, solid bars) in the right, ipsilateral to inoculation (I) and in the left, contralateral (C) hippocampus. Analysis has been performed 4 (A), 11 (B) and 25 days (C) after inoculation (DAI), i.e. 7, 14 and 28 days after pilocarpine SE. Data are presented as mean ± s.e.m. for 6 animals per group. *P < 0.05, **P < 0.01 versus naïve; ^●^P < 0.05, ^●●^P < 0.01 versus pilo-control vector. One-way ANOVA and post-hoc Newman-Keuls test. (D-F) Representative sections showing GFAP immunohistochemistry in the CA1 region of naïve rats (D), of pilocarpine rats killed 11 days after control vector injection (E) and of pilocarpine rats 11 days after FGF2-BDNF vector injection (F). The pattern of changes observed in CA1 was identical for the entire hippocampus. Horizontal bar = 50 μm.

The density of GFAP-positive cells in the hippocampus was significantly increased in pilocarpine-treated animals compared with naïve controls, an indication of reactive astrocytosis. In agreement with previous studies [18], this phenomenon was maximal during latency (7 days after SE) and then gradually decreased with time (Figure 1). In pilocarpine animals treated with TH-FGF2/0-BDNF, the density of GFAP-positive cells initially increased to about the same level as in those treated with the control vector (Figure 1A), but then decreased more rapidly, becoming significantly lower 14 days after SE (i.e. 11 days after vector inoculation; Figure 1B and 1E-F). However, this did not lead to a complete normalization of astrocytosis, but only to an acceleration of the process: 28 days after SE (25 days after vector inoculation) the expression of GFAP was identical in the two groups (Figure 1C).

**Figure 2:**
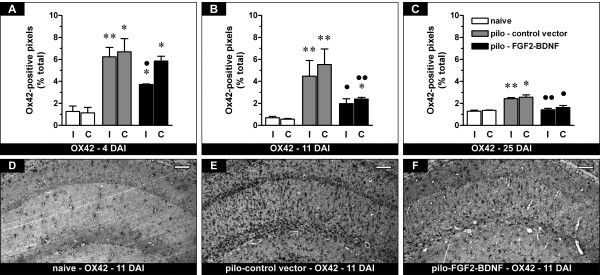
**Effects of FGF-2 and BDNF on hippocampal Ox42-positive cells (putative microglia)**. (A-C) Time course of the alterations in Ox42 expression, expressed as percent of Ox42-positive pixels. See Figure 1 for details. Data are presented as mean ± s.e.m. for 6 animals per group. *P < 0.05, **P > 0.01 versus naïve; ^●^P < 0.05, ^●●^P < 0.01 versus pilo-control vector. One-way ANOVA and post-hoc Newman-Keuls test. (D-F) Representative sections showing Ox42 immunohistochemistry in the CA1 region of naïve rats (D), of pilocarpine rats killed 11 days after control vector injection (E) and of pilocarpine rats 11 days after FGF2-BDNF vector injection (F). The pattern of changes observed in CA1 was identical for the entire hippocampus. Horizontal bar = 50 μm.

We then measured the density of microglial cells using Ox42 immunohistochemistry. Similar to GFAP-positive cells, the density of Ox42-positive cells in the hippocampus was significantly increased in pilocarpine-treated animals compared with naïve controls, an indication of reactive microgliosis, and this phenomenon was maximal during latency (7 days after SE), then gradually decreased in time (Figure 2). In pilocarpine animals treated with TH-FGF2/0-BDNF, the density of Ox42-positive cells was initially reduced only in the injected hippocampus (Figure 2A), then bilaterally (Figure 2B and 2E-F), paralleling high level transgene expression [5]. Twenty-eight days after SE it was at baseline levels (Figure 2C).

**Figure 3:**
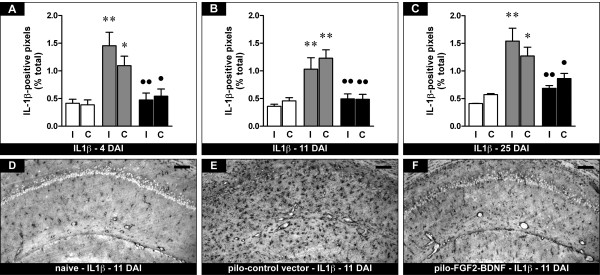
**Effects of FGF-2 and BDNF on hippocampal IL1β-positive cells**. (A-C) Time course of the alterations in IL1β expression, expressed as percent of IL1β-positive pixels. See Figure 1 for details. Data are presented as mean ± s.e.m. for 6 animals per group. *P < 0.05, **P < 0.01 versus naïve; ^●^P < 0.05, ^●●^P > 0.01 versus pilo-control vector. One-way ANOVA and post-hoc Newman-Keuls test. (D-F) Representative sections showing IL1β immunohistochemistry in the CA1 region of naïve rats (D), of pilocarpine rats killed 11 days after control vector injection (E) and of pilocarpine rats 11 days after FGF2-BDNF vector injection (F). The pattern of changes observed in CA1 was identical for the entire hippocampus. Horizontal bar = 50 μm.

In agreement with previous reports [19], IL-1β expression dramatically increased in pilocarpine-treated animals compared with naïve controls, in a long-lasting manner (Figure 3). In pilocarpine animals treated with the vector expressing FGF-2 and BDNF, this increase in IL-1β expression was prevented (Figure 3). The observation of a bilateral effect even at the earliest time-point, when contralateral transgene expression is still relatively low [5], indicates that low-level FGF-2 and BDNF supplementation may be sufficient to prevent IL-1β expression. Compared with pilocarpine animals, no change in any of the investigated parameters was observed in TH-FGF2/0-BDNF-treated animals in areas where transgene expression did not occur, such as neocortex (data not shown).

This study suggests that NTFs may affect neuroinflammation *in vivo*: the local, viral vector-mediated, supplementation of FGF-2 and BDNF in a lesioned, epileptogenic hippocampus attenuates various parameters of inflammation, including astrocytosis, microcytosis and IL-1β expression. The effect appears to be most prominent on IL-1β, whose expression is almost completely prevented, even at earliest time-point and for relatively low-level transgene expression.

Neuroinflammation appears to be highly implicated in epileptogenesis. Leukocyte-endothelium interaction may result in leukocyte extravasation and BBB permeabilization, favoring the induction of spontaneous seizures [10,13,20,21]. Integrin activation and BBB leakage are thought to exert a proepileptic role by modulating the release of cytokines and chemokines, which are known to modulate intercellular signaling within the CNS [22-25]. Very little is known regarding the possible modulation of these phenomena by NTFs. However, BDNF is upregulated in rodents after inflammatory challenges [26]. Also, BDNF and its receptor trkB are expressed in macrophages [27] and play autocrine and paracrine roles in the modulation of regeneration and angiogenesis following nerve injury [28]. Moreover, recent data suggest that pro-BDNF (the BDNF precursor) is a suppressing factor for macrophage migration and infiltration and may play a detrimental role after spinal cord injury [29].

CNS inflammation is associated with BBB breakdown, and BBB leakage has been implicated both in the induction of seizures and in the progression to epilepsy [6-9]. In addition, BBB opening leads by itself to epileptiform activity, mediated by exposure of astrocytes and neuronal cells to blood albumin and potassium ions, respectively [6,7]. In support of the results obtained from animal models, it has been shown that BBB disruption by intra-arterial injection of mannitol in human patients suffering from cerebral lymphoma induces focal motor seizures [9]. NTFs may be involved in preserving BBB integrity: intracerebroventricular administration of BDNF has been reported to significantly reduce BBB breakdown, brain edema formation, and cell/tissue injury [30]; moreover, FGF-2 also plays an important role in the regulation of BBB permeability in vivo [31].

BBB leakage, however, peaks about 4 days after pilocarpine-induced SE [32], while significant FGF-2 and BDNF overexpression is achieved only a few days after vector injection, i.e. about a week after SE [5,33]. Thus, effects on BBB may only partially explain the observed reduction in neuroinflammmatory signs. One alternative hypothesis is that FGF-2 and BDNF primarily reduce IL-1β expression, thereby attenuating astrocytosis and microgliosis [34,35]. This hypothesis is in line with the temporal sequence of the effects, wherein IL-1β effects appear earlier than those of GFAP and Ox42. Remarkably, the effect on IL-1β is observed before the occurrence of SRSs and, thus, cannot be secondary to their reduction.

In conclusion, the present study provides first evidence that NTF overexpression might modulate the brain inflammatory response that follows pilocarpine-induced SE. This effect may underlie the observed reduction in SRS frequency and, therefore, may represent an interesting therapeutic approach. Its molecular mechanism(s) will be explored in the future. Nonetheless, the concept that NTFs affect neuroinflammation in vivo may be highly relevant for the understanding of the epileptogenic process.

## Competing interests

The authors declare that they have no competing interests.

## Authors' contributions

PFF and MS conceived and designed the study; RB, SZ, BP, DR, FM, GNM, FO, EB PM and AM performed and analyzed the experiments. RB, PFF and MS drafted the manuscript. All authors have read and approved the final version of the manuscript.

## Supplementary Material

Additional file 1 Additional Material (Bovolenta et al.: *Hippocampal FGF-2 and BDNF Overexpression Attenuates Epileptogenesis-Associated Neuroinflammation and Reduces Spontaneous Recurrent Seizures*) Click here for file
