# Supplementary material for: Hippocampal FGF-2 and BDNF overexpression attenuates epileptogenesis-associated neuroinflammation and reduces spontaneous recurrent seizures
Source: J Neuroinflammation. 2010 Nov 18;7:81. doi: 10.1186/1742-2094-7-81 (PMC2993685; doi:10.1186/1742-2094-7-81)
Supplement: Additional file 1 — Additional Material (Bovolenta et al.: Hippocampal FGF-2 and BDNF Overexpression Attenuates Epileptogenesis-Associated Neuroinflammation and Reduces Spontaneous Recurrent Seizures) [file 1742-2094-7-81-S1.DOC]

**Additional Material**

**(Bovolenta et al.:** *Hippocampal FGF-2 and BDNF Overexpression Attenuates Epileptogenesis-Associated Neuroinflammation and Reduces Spontaneous Recurrent Seizures***)**

**Methods**

**Vectors**

Vectors were prepared as previously described [1,2]. A plasmid (pB410-BDNF) was constructed by introduction of the rat bdnf cDNA (1127 pb) [3] from pBluscript-BDNF into the HSV flank sequences of the previously described pB41 plasmid [4]. The *bdnf* cDNA was inserted, under the transcriptional control of the HSV-1 IE ICP0 promoter, into the XbaI sites of the pB41 plasmid, between the two UL41 HSV fragments [HSV genomic positions 90.145–91.631 and 92.230–93.858 [4]. pB410-BDNF was then recombined within the genome of the T0-LacZ viral vector using the previously described Pac-facilitated LacZ substitution method [4]. T0-LacZ is a replication-defective HSV-1 viral vector with the backbone of TH-LacZ (deletion of the three IE ICP4, ICP27, and ICP22; [5]), with the cDNA encoding LacZ inserted in the UL41 locus. The production of recombinant viruses was carried out using the standard calcium phosphate transfection procedure with 5 μg of viral DNA and 1 μg of linear pB410-BDNF. Transfection and isolation of the recombinant virus was performed in 7b cells, as previously described [4]. The recombinant virus T0-BDNF, containing the *bdnf* cDNA in the UL41 locus, was identified by isolation of a clear plaque phenotype after X-gal staining. This virus was purified by three rounds of limiting dilution and the presence of the transgene was verified by Southern blot analysis. Viral stocks of T0-BDNF were prepared and titrated using 7b cells.

The vector TH-FGF2/0-BDNF, containing FGF-2 in the *tk* (thymidine kinase) locus and BDNF in the UL41 locus, was created by genetically crossing the vectors TH-FGF2 [5] and T0-BDNF. 7b cells, plated in 60-mm Petri dishes, were infected with TH-FGF2 and T0-BDNF at a MOI of 3.0 and harvested 18 hours postinfection. The mixture of viruses derived from the co-infection was titrated, and the viral vector containing both genes was isolated by Southern blot screening. The TH-FGF2/0-BDNF virus was purified by three rounds of limiting dilution and expression of the transgenes confirmed by Western blot analysis. Purity of the vector was tested by verifying the absence of contaminating FGF-2 and BDNF proteins in each viral stock.

**Animals**

Male Sprague-Dawley rats (240–260 g; Harlan Italy) were used for all experiments. Animals were housed under standard conditions: constant temperature (22-24°C) and humidity (55-65%), 12 h dark-light cycle, free access to food and water. All efforts were made to minimize animal suffering. All procedures were carried out in accordance with guidelines by the European Community and national laws and policies (authorization from the Italian Ministry of Health n. 83/2009-B).

Pilocarpine was administered i.p. (300 mg/kg) 30 min after methyl-scopolamine (1 mg/kg s.c.), to minimize peripheral cholinergic effect. The rat’s behavior was observed for several hours thereafter. Within the first hour after injection, all animals developed seizures evolving into recurrent generalized convulsions (SE). SE was interrupted 2 hours after onset by administration of diazepam (10 mg/kg i.p.).

Three days after pilocarpine SE, under ketamine (87 mg/kg i.p.) and xylazine (13 mg/kg i.p.) anesthesia, a borosilicate glass needle connected to a perfusion pump was implanted in the right dorsal hippocampus (coordinates: 1.5 mm lateral and 1.7 mm posterior to bregma, 3.0 mm deep from dura). A total of 1.6×10^6^ pfu of vector were injected in a volume of 2 μl at a flow rate of 0.1 μl/min [2]. Trypan blue (at the nontoxic concentration of 0.01%) was added to the vector solution to allow precise identification of the injection site [6]. Animals were sacrificed 4, 11, or 25 days after vector injection. Controls were control vector-injected or naïve rats.

**Immunohistochemistry**

Rats were sacrificed by decapitation after an anesthetic overdose. Their brains were rapidly removed, immersed in 10% formalin and then paraffin embedded. Successive 6 µm sections were cut across the entire dorsal hippocampus (233 sections, corresponding to approximately 1.4 mm; plates 39-46 [7]) and mounted onto polarized slides (Superfrost slides, Diapath). One every 58 of these sections (5 sections per animal) was stained for the markers below. Sections were dewaxed, rehydratated and unmasked using a commercially available kit (Unmasker, Diapath), according to the manufacturer’s instructions. For activated microglia and astrocytes, we employed the Dako Cytomation EnVision® + Dual Link System-HRP (DAB+) kit. After washing in PBS 1×, sections were incubated for 10 min, at room temperature, with Endogenous Enzyme Block to quench endogenous peroxydase activity. Subsequently, they were incubated with the primary antibody (rabbit polyclonal anti CD11 b/c, clone Ox42, 1:200 dilution, Novus Biologicals; or rabbit polyclonal anti-GFAP, 1:200, Sigma) at room temperature. After 30 minutes, slices were rinsed twice with PBS 1× and incubated for another 30 min with Labeled Polymer-HRP [Dako Cytomation EnVision® + Dual Link System-HRP (DAB+)]. Staining was completed by a 3 min incubation with 3,3’-diaminobenzidine (DAB) substrated-chromogen, resulting in a brown staining of the antigen-antibody complex. Finally, sections were mounted using a water-based mounting medium (Shur Mount™, TBS).

For IL1β, sections were unmasked as described above and, after incubation in H_2_O_2_ 0.3% for 15 min at room temperature, rapidly rinsed in distilled water and washed again in PBS. They were then incubated for 10 min with Ultra V Block (Ultra Vision Detection System; Lab Vision Corporation) at room temperature, to block nonspecific background. After overnight incubation at 4°C, in humid atmosphere, with the primary antibody (goat polyclonal anti-IL1β, 1:200; Santa Cruz Biotechnology, Inc.), sections were rinsed in PBS 1× and incubated at room temperature for 2 h with HRPO Swine anti-goat IgG (H+L) human/mouse adsorbed (Cedarlane Laboratories). The reaction product was detected using DAB (ImmPACT DAB, Vector Laboratories, Inc). Finally, sections were washed in PBS and mounted using Shur Mount™ (TBS). The specificity of immunolabeling was verified in all experiments by controls in which the primary antibody was omitted.

Image analysis was conducted using a Leica microscope (DMRA2, Leica). The expression levels of GFAP, Ox42 and IL1β were measured using a thresholding approach [8] by investigators that were blind for the group to which the rats belonged. Images of the hippocampus were captured using a Leica DFC300FX camera and transformed into gray levels. Using Photoshop CS2 (version 9.0.2), the mean ± standard deviation gray level was calculated in visually identified positive cells. The hippocampus was then cut out, and positive pixels identified by thresholding at the gray level corresponding to the mean plus two standard deviations. Using this approach, only those pixels that were significantly above background were selected. Data have been expressed as percent of positive pixels over total pixels in the selected area. This method has been validated by comparing data with those obtained counting GFAP-positive cells. As stated above, 4 regularly spaced sections have been examined for each animal. The mean percent of positive pixels was calculated in these 4 sections and used for statistical analysis. Statistical analysis was conducted using one-way ANOVA and post-hoc the Newman-Keuls test.

**Telemetry EEG and behavioral analysis**

Together with vector injection (3 days after SE), animals were implanted i.p. with a TA11CTA-F40 telemetry transmitter (Data Science International, DSI, USA) with subcutaneous electrodes leading to the sub-dura mater above the parietal cortex. Silicon coating of all the leads was peeled back to expose approximately 5 mm of the helical steel lead. The tip of negative and positive leads created an angle of approximately 90 degrees. These radiotelemetry devices continuously sense, process and transmit information from the animal to a data storing system. Seizures were assessed by video-monitoring of the animals, performed by means of Phenotyper cages (Noldus Information Technology, the Netherlands) and an acquisition system using telemetric technology (Dataquest® A.R.T. Data Acquisition 4.3 for telemetry systems, DSI). Behavioral analysis related to homecage exploration were automatically detected and calculated by the Ethovision XT system (Noldus), whereas behavioral alteration and convulsion scorings were performed by the means of the Observer (Noldus), a semi-automatized system for behavioral recording. The MPEG4 Encoder and The Observer XT, which connected directly with the cages, were the systems used to acquire video data. The telemetric and video system synchronized data were acquired simultaneously by a time code signal (corresponding to The Observer PC clock), which was sent continuously during acquisition from The Observer PC to the Dataquest ART PC. This time code was then recognized by the Observer software, allowing the synchronization of the videos with the physiological telemetric data. We recorded 24 h/day for 21 consecutive days, beginning 7 days after SE.

Seizures have been defined here as previously described [9,10]. The term “seizure” was used to indicate any electrically recorded seizure (EEG seizure), both non-convulsive or convulsive. Seizures were categorized as paroxysmal activity of high frequency (>5 Hz) lasting for more than 20 s and characterized by a 3-fold amplitude increment over baseline. Seizure severity was scored using the scale of Racine [11]: 1, chewing or mouth and facial movements; 2, head nodding; 3, forelimb clonus; 4, generalized seizure with rearing; 5, generalized seizure with rearing and falling. The behaviors during non-convulsive seizures were those of class 1 or 2. Seizure detection was performed both visually and by the means of the Observer XT (Noldus), a semi-automatized system for behavioral recording. All EEG recordings were examined for artifacts, and all seizures were confirmed by visual inspection. Seizure detection and single ictal events (<2 sec) were scored for each individual rat for the whole analysis period. Analysis was performed by two independent investigators that were blind for the group to which the rats belonged. In case of differential evaluation, data were reviewed together to reach a consensus.

**References**

1. Marconi P, Zucchini S, Berto E, Bozac A, Paradiso B, Bregola G, Grassi C, Volpi I, Argnani R, Marzola A, Manservigi R, Simonato M: **Effects of defective herpes simplex vectors expressing neurotrophic factors on the proliferation and differentiation of nervous cells in vivo.** *Gene Therapy* 2005, **12**:559-569.
2. Paradiso B, Marconi P, Zucchini S, Berto E, Binaschi A, Bozac A, Buzzi A, Mazzuferi M, Magri E, Navarro Mora G, Rodi D, Su T, Volpi I, Zanetti L, Marzola A, Manservigi R, Fabene PF, Simonato M: **Localized delivery of fibroblast growth factor-2 and brain-derived neurotrophic factor reduces spontaneous seizures in an epilepsy model.** *Proc Natl Acad Sci USA* 2009, **106**:7191-7196.
3. Maisonpierre PC, Le Beau MM, Espinosa R 3rd, Ip NY, Belluscio L, de la Monte SM, Squinto S, Furth ME, Yancopoulos GD: **Human and rat brain-derived neurotrophic factor and neurotrophin-3: gene structures, distributions, and chromosomal localizations.** *Genomics* 1991, **10**:558-568.
4. Krisky DM, Marconi PC, Oligino T, Rouse RJ, Fink DJ, Glorioso JC: **Rapid method for construction of recombinant HSV gene transfer vectors.** *Gene Ther* 1997, **4**:1120-1125.
5. Marconi P, Simonato M, Zucchini S, Bregola G, Argnani R, Krisky D, Glorioso JC, Manservigi R: **Replication-defective herpes simplex virus vectors for neurotrophic factor gene transfer in vitro and in vivo.** *Gene Therapy* 1999, **6**:904-912.
6. Parent JM, Valentin VV, Lowenstein DH: **Prolonged seizures increase proliferating neuroblasts in the adult rat subventricular zone-olfactory bulb pathway.** *J Neurosci* 2002, **22**:3174-3188.
7. Pellegrino LJ, Pellegrino AS, Cushman AJ: *A stereotaxic atlas of the rat brain*. New York: Plenum Press; 1979.
8. Mazzuferi M, Palma E, Martinello K, Maiolino F, Roseti C, Fucile S, Fabene PF, Schio F, Pellitteri M, Sperk G, Miledi R, Eusebi F, Simonato M: **Enhancement of GABA(A)-current run-down in the hippocampus occurs at the first spontaneous seizure in a model of temporal lobe epilepsy.** *Proc Natl Acad Sci USA* 2010, **107**:3180-3185.
9. Williams PA, White AM, Clark S, Ferraro DJ, Swiercz W, Staley KJ, Dudek FE: **Development of spontaneous recurrent seizures after kainate-induced status epilepticus.** *J Neurosci* 2009, **29**:2103-2112.
10. Paradiso B, Zucchini S, Su T, Bovolenta R, Berto E, Marconi P, Marzola A, Navarro Mora G, Fabene PF, Simonato M: **Localized overexpression of FGF-2 and BDNF in hippocampus reduces mossy fiber sprouting and spontaneous seizures up to four weeks after pilocarpine-induced status epilepticus.** *Epilepsia*, in press.
11. Racine RJ: **Modification of seizure activity by electrical stimulation. II. Motor seizure.** *Electroencephalogr Clin Neurophysiol* 1972, **32**:281-294.
